# The Nuclear Receptor Seven Up Regulates Genes Involved in Immunity and Xenobiotic Response in the Adult *Drosophila* Female Fat Body

**DOI:** 10.1534/g3.120.401745

**Published:** 2020-10-21

**Authors:** Lesley N. Weaver, Daniela Drummond-Barbosa

**Affiliations:** Department of Biochemistry and Molecular Biology, Bloomberg School of Public Health, Johns Hopkins University, Baltimore, MD 21205

**Keywords:** *Drosophila*, fat body, Seven up, oogenesis, xenobiotics, immunity

## Abstract

The physiology of organisms depends on inter-organ communication in response to changes in the environment. Nuclear receptors are broadly expressed transcription factors that respond to circulating molecules to control many biological processes, including immunity, detoxification, and reproduction. Although the tissue-intrinsic roles of nuclear receptors in reproduction have been extensively studied, there is increasing evidence that nuclear receptor signaling in peripheral tissues can also influence oogenesis. We previously showed that the *Drosophila* nuclear receptor Seven up (Svp) is required in the adult fat body to regulate distinct steps of oogenesis; however, the relevant downstream targets of Svp remain unknown. Here, we took an RNA sequencing approach to identify candidate Svp targets specifically in the adult female fat body that might mediate this response. *svp* knockdown in the adult female fat body significantly downregulated immune genes involved in the first line of pathogen defense, suggesting a role for Svp in stimulating early immunity. In addition, we found that Svp transcriptionally regulates genes involved in each step of the xenobiotic detoxification response. Based on these findings, we propose a testable model in which Svp functions in the adult female fat body to stimulate early defense against pathogens and facilitate detoxification as part of its mechanisms to promote oogenesis.

Nuclear receptors are evolutionarily conserved systemic physiology sensors that act as transcriptional regulators throughout the body to control a diverse array of biological processes, including female reproduction (Mouzat *et al.* 2013; Mazaira *et al.* 2018). For example, Estrogen Receptor global knockout results in elevated steroid synthesis, reducing the number of growing follicles and preventing meiotic oocyte progression (Lubahn *et al.* 1993; Liu *et al.* 2017). Global knockout of Liver X Receptors disrupts meiosis resumption and reduces mammalian fertility (Mouzat *et al.* 2013), whereas COUP-TFs are required in the mammalian uterine muscle for placenta formation and embryo implantation (Petit *et al.* 2007; Zheng *et al.* 2010). However, how nuclear receptor function in specific peripheral tissues influences oogenesis in adult females is understudied.

The *Drosophila* ovary is an ideal model to investigate how nuclear receptor action in peripheral tissues remotely influences oogenesis. The *Drosophila* ovary is composed of 16-20 ovarioles, and each ovariole has an anterior germarium followed by chronologically arranged developing follicles. Each germarium houses two to three germline stem cells (GSCs) in a specialized niche, and GSCs divide asymmetrically to self-renew and produce a cystoblast that undergoes four incomplete mitotic divisions to form a 16-cell cyst (one oocyte plus 15 supporting nurse cells). Follicle cells surround the 16-cell cyst to form a follicle that buds from the germarium and develops through 14 stages of oogenesis to form a mature oocyte (Figure 1A,B) (Greenspan *et al.* 2015; Laws and Drummond-Barbosa 2017). Many steps of oogenesis, including maintenance of GSCs, early germline cyst survival, vitellogenesis (*i.e.*, yolk uptake), and ovulation, are highly sensitive to physiological inputs (Drummond-Barbosa 2019). Notably, each of these processes has been shown to be controlled by nuclear receptors [reviewed in (Ables and Drummond-Barbosa 2017; Laws and Drummond-Barbosa 2017)].

**Figure 1:**
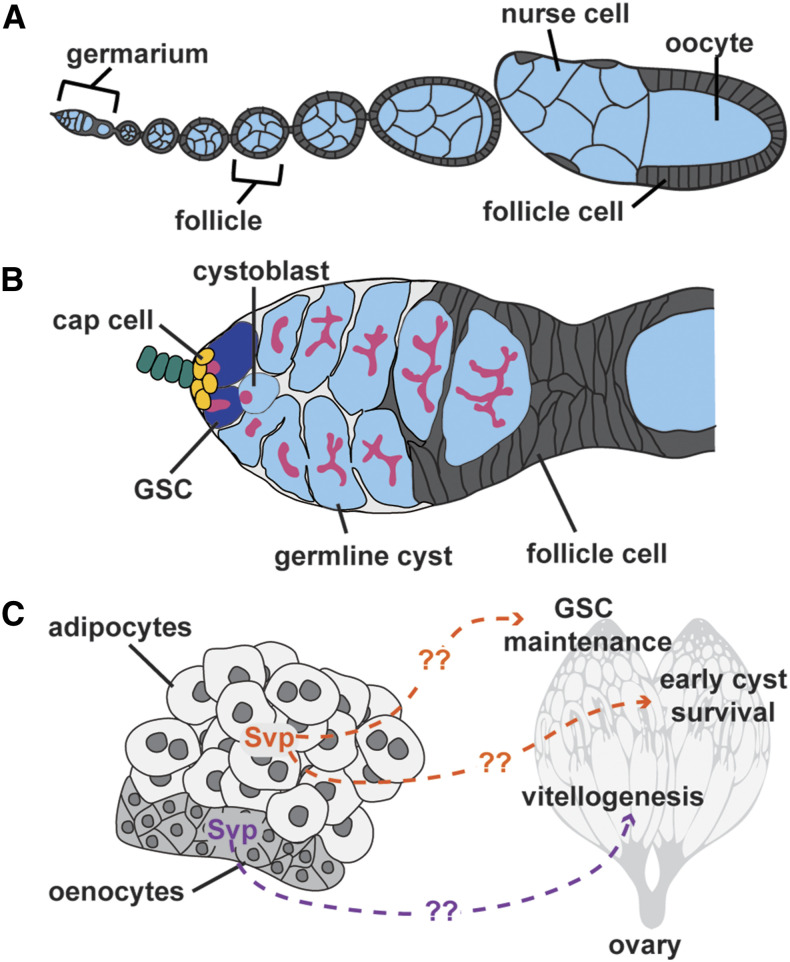
Svp functions through unknown downstream targets in the adult female fat body to regulate oogenesis. (A) *Drosophila* ovariole showing an anterior germarium followed by progressively older follicles. Each follicle represents a 16-cell germline cyst (one oocyte and 15 supporting nurse cells; light blue) surrounded by follicle cells (gray). (B) Germarium showing 2-3 germline stem cells (GSCs; dark blue) in a niche composed of somatic cells, including cap cells (yellow). GSCs give rise to cystoblasts that divide to form 16-cell cysts. Germline cysts are surrounded by follicle cells (gray) to bud from the germarium as a new follicle. (C) Svp is required specifically in adult female adipocytes to promote GSC maintenance and early germline cyst survival, and in oenocytes for survival of vitellogenic follicles.

Nuclear receptors have been shown to control *Drosophila* oogenesis through intrinsic and extrinsic mechanisms (Ables and Drummond-Barbosa 2017). The best characterized *Drosophila* nuclear receptor, EcR (FBgn0000546, FXR/LXR homolog), is required in ovarian cell types for regulation of GSC maintenance, early germline cyst survival and development, oocyte meiosis entry, and lipid uptake in later follicles (Ables and Drummond-Barbosa 2010; König *et al.* 2011; Morris and Spradling 2012; Sieber and Spradling 2015). E78 (FBgn0004865, potential PPAR homolog) is required for follicle development/survival (Ables and Drummond-Barbosa 2010), E75 (FBgn0000568, potential REV-ERB homolog) is cell autonomously required for vitellogenesis (Buszczak *et al.* 1999; Ables and Drummond-Barbosa 2010), and HR39 (FBgn0261239, LRH1 homolog) and Ftz-F1 (FBgn0001078, SF1 homolog) are required for ovulation (Sun and Spradling 2013; Knapp *et al.* 2020). In addition to having these ovary-intrinsic roles, nuclear receptors can also function in peripheral tissues to control oogenesis, as recent studies show (Sieber and Spradling 2015; Weaver and Drummond-Barbosa 2019). For example, EcR is required in the central nervous system to regulate female feeding behavior and thereby support egg production (Sieber and Spradling 2015). More recently, we showed that the nuclear receptor Svp is required in adult female adipocytes and hepatocyte-like oenocytes (collectively referred to as the fat body herein) to regulate distinct steps of oogenesis (Weaver and Drummond-Barbosa 2019). Specifically, Svp (FBgn0003651) is required in adipocytes to control GSC maintenance and early germline cyst survival, whereas Svp is required in oenocytes for survival of vitellogenic follicles (Figure 1C) (Weaver and Drummond-Barbosa 2019). However, the mechanisms underlying how nuclear receptor activity in peripheral tissues regulates oogenesis are not well understood.

The *Drosophila* fat body is a major endocrine organ with energy-intensive metabolic and immune roles (Arrese and Soulages 2010), raising many possibilities as to how Svp activity in adipocytes and oenocytes might remotely control oogenesis. Multiple studies have explored the nutrient sensing and metabolic roles of the fat body in regulating larval growth (Colombani *et al.* 2003; Hennig *et al.* 2006), in mobilizing lipids in response to starvation (Chatterjee *et al.* 2014), in controlling lifespan (Giannakou *et al.* 2004; Hwangbo *et al.* 2004), and in reproduction (Armstrong *et al.* 2014; Matsuoka *et al.* 2017; Armstrong and Drummond-Barbosa 2018). The function of the fat body as a major immune-responsive tissue has also been characterized (Lemaitre and Hoffmann 2007). In response to infection, the fat body activates nuclear factor-κB (NF-κB) signaling to produce and secrete antimicrobial peptides (AMPs) into the hemolymph (Lemaitre and Hoffmann 2007; Buchon *et al.* 2014; Roth *et al.* 2018; Suzawa *et al.* 2019). There is also evidence that the fat body acts as a detoxification tissue based on the expression of members of the Cytochrome P450 (Cyp450) superfamily of monooxygenases, which are enzymes involved in metabolizing foreign substances and drugs (Feyereisen 1999; Chung *et al.* 2009) and implicated in resistance to insecticides (Terhzaz *et al.* 2015). In this study, we used transcriptomics to identify candidate target genes downstream of Svp in the adult female fat body and generate hypotheses for future investigation into the molecular mechanisms underlying Svp control of oogenesis. Specifically, we took an RNA sequencing approach to compare the transcriptome of fat bodies from adult females with *svp* RNAi knockdown to that of control RNAi female fat bodies. We found that *svp* knockdown significantly reduces the expression levels of genes involved in the first line of defense against pathogens. In addition, Svp targets are significantly enriched for genes involved in xenobiotic detoxification responses. We propose a model according to which Svp normally functions in the adult female fat body to stimulate early immunity (and prevent later activation of the immune deficiency pathway) and to neutralize toxic compounds to facilitate their elimination from the body, thereby promoting optimal conditions for oogenesis.

## Materials And Methods

### Drosophila strains and culture conditions

*Drosophila* strains and cultures were maintained on medium containing 58 g/ml molasses, 46.5 g/ml yellow cornmeal, 4.7 g/ml agar, 17.4 g/ml active dry yeast, 0.1% tegosept, and 7.25 mM Propionic Acid at 22-25°. The previously described Gal4 lines, adipocyte-specific *3.1Lsp2-Gal4* (Lazareva *et al.* 2007; Armstrong *et al.* 2014) and *PromE800-Gal4* (Billeter *et al.* 2009; Weaver and Drummond-Barbosa 2019), along with the previously described temperature-sensitive *tub-Gal80^ts^* transgene (Mcguire *et al.* 2003), were recombined by standard crosses to generate the *PromE800-Gal4 tubGal80^ts^; 3.1Lsp2-Gal4* double driver specifically targeting both oenocytes and adipocytes with temporal control. *UAS-Luc^JF01355^* (Matsuoka *et al.* 2017) was obtained from the Bloomington *Drosophila* Stock Center and *UAS-svp*^*GD1546*^ was obtained from the Vienna *Drosophila* Resource Center (VDRC). Balancer chromosomes and other genetic elements are described in Flybase (www.flybase.org).

### Tissue-specific RNAi

Females of genotypes *y w; PromE800-Gal4 tubGal80^ts^; 3.1Lsp2-Gal4/UAS-hairpin* (for adult fat body-specific RNAi) were raised at 18° [the permissive temperature for Gal80^ts^ (Mcguire *et al.* 2003)] to prevent RNAi induction during development. Zero- to 2-day-old females were maintained at 18° for 3 days with *y w* males, and then switched to 29° (the restrictive temperature for Gal80^ts^) for 5 days to induce RNAi in the adult fat body (*i.e.*, in both adipocytes and oenocytes). We chose this time point of *svp* RNAi in part because knockdown of *svp* specifically in adult oenocytes decreases egg production after 5 days (Weaver and Drummond-Barbosa 2019). We also note that, although reduced GSC numbers are only observed after 7-10 days of *svp* knockdown (Weaver and Drummond-Barbosa 2019), any changes in gene expression resulting from decreased Svp activity that could be causally involved in the increased rate of GSC loss would necessarily precede any observable decrease in GSC numbers. *UAS-Luc^JF01355^* was used as an RNAi control. For all conditions, medium was supplemented with wet yeast paste.

### RNA isolation

Abdominal carcasses from 100 females of each genotype were dissected in Grace’s medium supplemented with 10% fetal bovine serum (FBS; Sigma). Fat body cells were dissociated from abdominal carcasses with 500 µl dissociation buffer [0.5% Trypsin (Sigma) plus 1 mg/ml collagenase (Sigma) in 1x PBS] per 50 carcasses for 30 min at room temperature. Samples were gently agitated every 5 min to facilitate separation of cells from the cuticle. 500 µl of Grace’s media plus 10% FBS was added to stop the enzymatic reaction and supernatants were placed in new tubes. Carcasses were rinsed with Grace’s medium plus 10% FBS and the two supernatants per genotype were combined. Dissociated cells were centrifuged at 3.3 rpm for 5 min at room temperature. Supernatants were removed and cells were immediately lysed in 250 µl lysis buffer from the RNAqueous-4PCR DNA-free RNA isolation for RT-PCR kit (Ambion). RNA was extracted from all samples following the manufacturer’s instructions. Three independent experiments were performed for RNA sequencing and RT-qPCR analysis.

### RT-qPCR

RNA from abdominal carcasses was extracted as described above. cDNA was synthesized from 500 ng of total RNA for each sample using Supercript II Reverse Transcriptase (ThermoFisher) according to the manufacturer’s instructions. Table S1 lists all primers used in this study. PowerUp SYBR Green Master Mix (ThermoFisher) was used for quantitative PCR. The reactions for three independent biological replicates were performed in triplicate using LightCycler 96 (Roche). Amplification fluorescence threshold was determined by LightCycler 96 software, and ΔΔCT were calculated using Microsoft Excel. *Rp49* transcript levels were used as a reference. Fold change of transcript levels were calculated using the equation 2^-ΔΔCt^ (Microsoft Excel).

### RNA sequencing and data analysis

cDNA library construction, Illumina sequencing, and differential expression analysis was performed by Novogene Bioinformatics Technology Co., Ltd (Beijing, China). The cDNA libraries were prepared using the NEBNext Ultra RNA Library Prep Kit for Illumina (New England Biolabs) according to the manufacturer’s instructions. The cDNA library for each sample was quality assessed using an Agilent Bioanalyzer 2100, and library preparations were sequenced on a NovaSeq6000 platform with PE150 read lengths.

Reads obtained from sequencing were aligned to the *D. melanogaster* reference genome using the TopHat read alignment tool (Trapnell *et al.* 2009) for each of the sequencing datasets. The reference sequences were downloaded from the Ensembl project website (useast.ensembl.org). TopHat alignments were used to generate read counts for each gene using HTSeq (Anders *et al.* 2015), which were subsequently used to generate the differential expression results using the DESeq2 R package (Anders *et al.* 2015). Gene ontology (GO) enrichment of differentially expressed genes was analyzed by the clusterProfiler R package (Yu *et al.* 2012). Enriched genes with a corrected *P* value of less than 0.05 were considered significant.

### Data availability

*Drosophila* strains are available upon request. The data and analyses in this paper are described in the main figures. The raw data and processed data files are available through the NCBI GEO accession number GSE159703 and are also provided as supplemental tables. Supplemental material available at figshare: https://doi.org/10.25387/g3.13122728.

## Results And Discussion

### Expression levels of 132 transcripts are altered when svp is knocked down in the fat body

**Figure 2:**
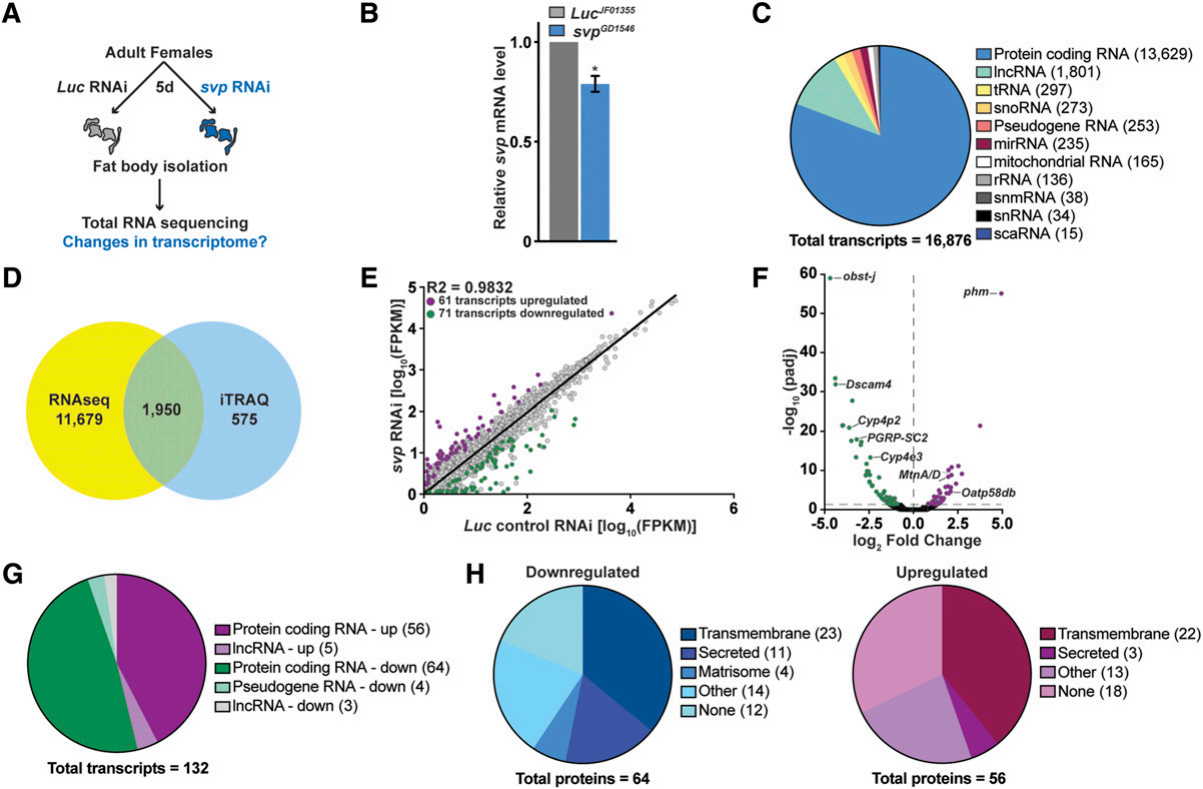
*svp* knockdown in the adult female fat body differentially regulates 132 genes. (A) Schematic of *svp* RNAi and RNA isolation in adult females. (B) RT-qPCR analysis of *svp* from fat bodies of females at five days of fat body *Luc* control or *svp* RNAi showing the mean±SEM. *P <0.05. (C) Pie chart showing the types of transcripts identified in the adult female fat body by RNA sequencing. (D) Venn diagram comparing the number of genes identified by RNA sequencing to the number of genes identified by iTRAQ proteomic analysis (Matsuoka *et al.* 2017) in adult female fat bodies. (E) Scatter plot of normalized transcript abundance in FPKM (fragments per kilobase of transcript per million mapped reads) of control vs *svp* knockdown fat bodies. Significantly upregulated genes, purple; significantly downregulated genes, green; unchanged, gray. (F) Volcano plot of differentially expressed genes graphing the statistical significance [-log_10_(padj)] against the magnitude of differential expression (log_2_ Fold Change). The horizontal dotted line represents *P*=0.05, such that all points above that line are considered statistically significant. (G) Classification of genes corresponding to significantly changed transcript levels in *svp* RNAi compared to control RNAi. (H) Pie charts of protein-coding gene classifications based on GLAD analysis for significantly downregulated (blue) and upregulated (purple) genes.

We previously showed that Svp is required in the adult female fat body to regulate distinct aspects of oogenesis (Weaver and Drummond-Barbosa 2019); however, the downstream factors mediating those effects are unknown. To identify downstream targets of Svp in the adult female fat body, we performed RNA sequencing analysis of fat bodies from fat body-specific *Luc* control RNAi and *svp* RNAi females (Figure 2A). We knocked down *svp* in the entire fat body of adult females for 5 days, using the combined adipocyte-specific *3.1Lsp2-Gal4* (Lazareva *et al.* 2007; Armstrong *et al.* 2014) and oenocyte-specific *PromE800-Gal4* (Billeter *et al.* 2009; Weaver and Drummond-Barbosa 2019) drivers with *tub-Gal80^ts^* (Mcguire *et al.* 2003), prior to fat body dissections and RNA sequencing performed in triplicate. RT-qPCR analysis showed that *svp* transcript levels were significantly decreased by 21% in *svp* knockdown relative to control fat bodies (Figure 2B). [We note that this modest knockdown is sufficient to cause significant changes in oogenesis (Weaver and Drummond-Barbosa 2019), consistent with physiological regulators being highly sensitive to environmental and physiological fluctuations. In fact, we previously showed that similarly modest changes in the levels of single amino acid transporters in adipocytes also lead to increased GSC loss (Armstrong *et al.* 2014)]. RNA sequencing produced an average of 38,389,394 reads across the six sequencing libraries, ranging from 35.1 to 43.3 million reads per sample (Table 1), of which an average of 96% were mapped to the *Drosophila melanogaster* genome. Based on the analysis of those reads, over 16,000 transcripts were identified in the adult female fat body, with the majority (81%) representing protein-coding genes (Figure 2C; Table S2). In addition to protein-coding genes, our RNA sequencing analysis also identified transcripts for mitochondrial genes, long non-coding RNAs (lncRNAs), small nucleolar RNAs (snoRNAs), microRNAs (miRNAs), and additional RNA subtypes (Figure 2C). Our samples were enriched for mRNAs using oligo(dT) beads for poly(A) selection; however, this method does not completely eliminate non-coding RNAs, especially those containing poly(A) tails (Cai *et al.* 2004; Wilhelm *et al.* 2008; Cabili *et al.* 2011). Comparison of the identified protein-coding genes with our previous iTRAQ adult female fat body proteomic data (Matsuoka *et al.* 2017) revealed almost 2,000 common genes between the two datasets (Figure 2D; Table S3), representing ∼75% of the total proteins we identified by iTRAQ (Matsuoka *et al.* 2017). These results suggest that our transcriptomic and proteomic approaches reproducibly identified many of the genes expressed in the adult female fat body.

**Table 1:** Total number of mapped reads for each RNA sequencing library

Sample	# of Reads	# of Mapped Reads	% Mapped
*Luc* control, Rep 1	38,922,918	38,040,044	97.73%
*Luc* control, Rep 2	40,188,640	38,345,558	95.41%
*Luc* control, Rep 3	41,454,476	39,698,160	95.76%
*svp* RNAi, Rep 1	41,454,476	43,317,932	96.71%
*svp* RNAi, Rep 2	36,380,868	35,132,654	96.57%
*svp* RNAi, Rep 3	38,922,918	37,253,098	95.71%

Differential expression analysis identified 132 genes with significantly altered transcript levels between the *Luc* control RNAi and *svp* RNAi groups (Figure 2E,F). Of those genes, 71 were downregulated (indicating genes normally positively regulated by Svp; Table S4), whereas 61 genes were significantly upregulated (indicating genes normally negatively regulated by Svp; Table S5). Of the upregulated genes, 92% were protein-coding genes and the remainder were identified as lncRNAs (Figure 2G). In addition, analysis using the gene group resource GLAD (Hu *et al.* 2015) classified most upregulated genes as encoding proteins that are either transmembrane, secreted, part of the matrisome, or do not belong to a specific category (referred to as “None,” Figure 2H). A small subset of genes were singly classified as encoding mitochondrial proteins, serine proteases, phosphatases, or methyltransferases (Figure 2H; listed as “Other”). Interestingly, a small subset of downregulated genes was identified as encoding proteins that are part of the “matrisome,” which form or remodel the extracellular matrix (Davis *et al.* 2019) (Figure 2H). The majority of downregulated genes were identified as protein-coding genes (Figure 2G), with most of those classified as encoding transmembrane or secreted proteins by GLAD analysis (Figure 2H). Similar to the case for upregulated genes, genes encoding serine proteases, kinases, and methyltransferases (referred to as “Other”) were also identified among downregulated genes (Figure 2H).

Multiple nuclear receptors have known roles in adipocytes in regulating lipid metabolism or controlling insulin secretion/sensitivity in mammals and *Drosophila* [reviewed in (King-Jones and Thummel 2005; Liu *et al.* 2015)]. For example, *HNF4A*-deficient mice are glucose intolerant and display impaired glucose-stimulated insulin secretion (Gupta *et al.* 2005), whereas mice heterozygous for the Svp homolog COUP-TFII have reduced adiposity and increased insulin sensitivity (Li *et al.* 2009). In *Drosophila*, *Hnf4* (FBgn0004914) is required for lipid mobilization and β-oxidation in response to starvation in larvae (Palanker *et al.* 2009), whereas loss of *svp* in the larval fat body impairs lipid turnover and insulin signaling (Musselman *et al.* 2018). Surprisingly, however, the list of genes with significantly altered expression in the *svp* RNAi fat body was not enriched for genes encoding metabolic proteins based on GLAD or Gene Ontology (GO) analysis. In fact, genes encoding members of the insulin signaling, acetyl-CoA production, or β-oxidation pathways were not differentially expressed in *svp* knockdown fat bodies compared to RNAi controls (Table S2). These results suggest that, in stark contrast to known roles of nuclear receptors in regulating metabolic processes (*e.g.*, HNF4, ERR, HR96) (Palanker *et al.* 2009; Sieber and Thummel 2009; Tennessen *et al.* 2011), Svp activity in the adult female fat body regulates genes that control other biological functions instead.

### Svp induces genes involved in the early defense against microorganisms in the adult female fat body

**Figure 3:**
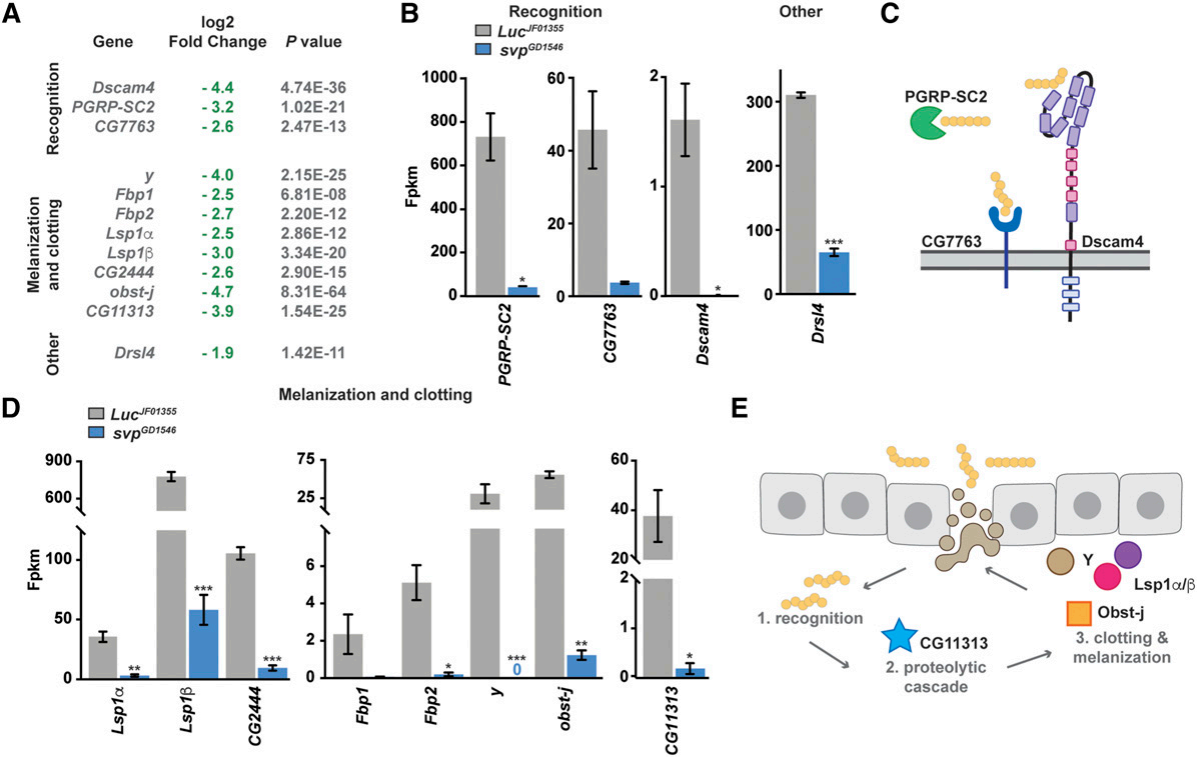
Knockdown of *svp* in adult female fat bodies reduces expression of genes involved in bacterial recognition, melanization, and clotting. (A) Classification of genes significantly downregulated with a log2 fold change of -1.5 or higher. (B) Quantification of normalized transcript expression (FPKM) for significantly downregulated recognition genes in control and *svp* RNAi fat bodies. Data shown as mean±SEM. *P <0.05; ***P <0.001, two-tailed Student’s *t*-test. (C) Cartoon of peptidoglycan recognition proteins (bound to peptidoglycan present in cell walls of most bacteria, yellow circles), whose transcripts are significantly downregulated by *svp* RNAi. (D) Quantification of normalized transcript expression (FPKM) for significantly downregulated melanization and clotting genes in control and *svp* RNAi fat bodies. Data shown as mean±SEM. *P <0.05; **P <0.01; ***P <0.001, two-tailed Student’s *t*-test. (E) Simplified cartoon of the melanization and clotting cascade in response injury. Proteins encoded by significantly downregulated genes in the absence of *svp* are included.

To narrow down our list of candidate Svp targets, we first determined whether genes whose fat body expression levels were altered with a log2 fold change of at least ±1.5 when *svp* was knocked down in the adult female fat body belonged to common categories or pathways. Interestingly, we found that Svp positively regulates genes encoding proteins with roles in immunity, including those involved in particle recognition (*e.g.*, *PGRP-SC2*, FBgn0043575), melanization and clotting (*e.g.*, *y*, FBgn0004034), as well as anti-microbial peptides (*e.g.*, *Drsl4*, FBgn0052282) (Figure 3A). For example, three microbial recognition proteins, which recognize bacteria through pattern recognition receptors and act upstream of the Toll and immune deficiency (IMD) pathways (Lemaitre and Hoffmann 2007), were significantly downregulated when *svp* was knocked down in the adult female fat body (Figure 3A-C). Peptidoglycan recognition protein (PGRP-SC2), whose transcript was downregulated with a log2 fold change of -3.2 (*i.e.*, ∼9-fold) by *svp* RNAi (Figure 3A,B), is a secreted protein that scavenges bacteria in the hemolymph to prevent activation of the IMD pathway (Figure 3C) (Bischoff *et al.* 2006; Paredes *et al.* 2011). The immunoglobulin-superfamily receptor Down syndrome adhesion molecule 4 (Dscam4, FBgn0263219), whose transcript was downregulated with a log2 fold change of -4.4 by *svp* RNAi (Figure 3A,B), is a transmembrane protein required for phagocytic uptake of bacteria (Figure 3C) (Watson *et al.* 2005). *CG7763* (FBgn0040503) was downregulated with a log2 fold change of -2.6 (Figure 3A,B) and encodes a predicted member of the C-type lectin family, which has been implicated in recognition of lipopolysaccharide [a component of gram negative bacterial cell walls (Zhang *et al.* 2013)] (Figure 3C) (Theopold *et al.* 1999). *Drsl4*, which encodes an antifungal peptide with homology to *Drs* (Jiggins and Kim 2005), was also significantly downregulated (log2 fold change of -1.9; Figure 3A,B) in the *svp* knockdown adult female fat body.

In addition to the responses involved in recognizing foreign pathogens upon injury and infection, another important immune defense is proper wound healing (Theopold *et al.* 2004). At the site of injury, a melanization cascade is initiated; melanization requires recognition of a pathogen (through pattern recognition receptors) followed by activation of phenoloxidase by serine proteases to ultimately produce melanin, which crosslinks and encapsulates microbes (Bidla *et al.* 2005; Binggeli *et al.* 2014). In addition to melanization, clotting is critical for limiting hemolymph loss as well as for physically immobilizing bacteria (Goto *et al.* 2003; Theopold *et al.* 2004). Our RNA sequencing analysis identified eight genes with roles in promoting melanization and clotting that are positively regulated by Svp in the adult female fat body (Figure 3D,F). For example, the *y* gene, downregulated with a log2 fold change of -4 (*i.e.*, 16-fold) by *svp* RNAi (Figure 3D,E), is required for melanin production (Biessmann 1985; Whittkopp *et al.* 2002). Of the clotting genes regulated by Svp (Figure 3D,E), *Fbp1* (FBgn0000639) encodes a known clotting factor originally isolated from larval hemolymph clots (Scherfer *et al.* 2004), and *obst-j* (FBgn0036940) encodes a chitin-binding protein that is upregulated in response to bacterial infection (Rynes *et al.* 2012). *Lsp1α* (FBgn0002562) and *Lsp1β* (FBgn0002563) encode predicted hemocyanins [proteins with known roles in regulating immunity (Coates and Nairn 2014)] and were also isolated from larval hemolymph clots (Scherfer *et al.* 2004). *CG11313* (FBgn0039798), which is downregulated with a log2 fold change of -3.9 upon *svp* RNAi (Figure 3D,E), encodes a predicted serine protease that has potential roles in immunity (Karlsson *et al.* 2004).

Nuclear receptors have reported roles in regulating immunity (Glass and Ogawa 2006; Glass and Saijo 2010). For example, mammalian PPARγ is required for wound healing and promotes the transcription of anti-inflammatory genes in macrophages, whereas LXR is important for macrophage inflammatory responses [reviewed in (Leopold Wager *et al.* 2019)]. Immune response genes are differentially regulated in *Drosophila* mutants for *Hr3* (FBgn0000448) (Ruaud *et al.* 2010), *Hnf4* (Barry and Thummel 2016), and *Hr4* (FBgn0264562) (King-Jones *et al.* 2005), suggesting that nuclear receptors also have immune response roles in invertebrates. However, only the developmental and metabolic roles of these nuclear receptors have been extensively studied [reviewed in (King-Jones and Thummel 2005)]. Our RNA sequencing analysis suggests that Svp normally functions in the adult female fat body as the first line of defense against infections (*e.g.*, microbial and viral recognition, peptidoglycan scavenging, and clotting/melanization upon wounding), potentially preventing activation of the IMD pathway (Lemaitre and Hoffmann 2007). Although we did not observe an increase in anti-microbial peptide synthesis associated with IMD activation (Lee and Lee 2018), it is possible that our time window of *svp* knockdown is insufficiently long. In fact, a previous study in the larval fat body also showed that Svp regulates immune response genes including *PGRP-SC2* and additional anti-microbial peptides (Musselman *et al.* 2018); however, Svp negatively regulates these genes in this context (Musselman *et al.* 2018). The seemingly opposite regulatory roles of Svp in larvae compared to adults could potentially be due to the organism’s distinct physiology at different developmental stages. For example, in larvae, nutrient availability plays a crucial role in growth and timing of metamorphosis (Colombani *et al.* 2003); by contrast, adult females have completed development but require large amounts of nutrients and energy to support egg production (Laws and Drummond-Barbosa 2017). The larval fat body is also remodeled during metamorphosis (Nelliot *et al.* 2006), which might alter the mechanisms linking immunity to physiology. Indeed, the nuclear receptor HNF4 was recently shown to be required in oenocytes for the conversion of larval lipid reservoirs into hydrocarbons for cuticular waterproofing in adults (Storelli *et al.* 2019), suggesting that the fat body is subject to different physiological demands depending on the developmental stage. Collectively, our results suggest that Svp may function in the adult female fat body to regulate genes that scavenge and eliminate foreign pathogens as a mechanism to prevent activation of the IMD pathway [which is an energy-intensive process (Buttgereit *et al.* 2000; Davoodi *et al.* 2019)], thereby freeing up resources to support reproduction in adult females.

### Svp modulates the transcription of genes involved in xenobiotic responses

**Figure 4:**
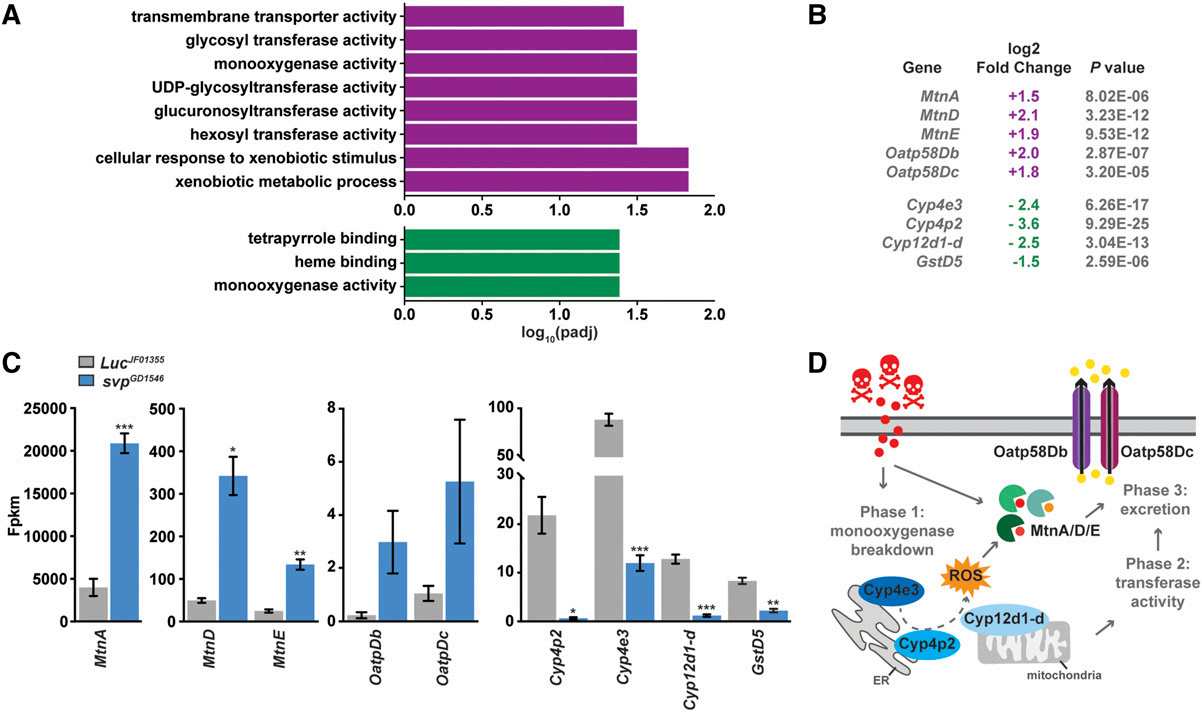
Differentially expressed transcripts are significantly enriched for genes involved in xenobiotic responses. (A) Gene ontology (GO) analysis of significantly upregulated (purple) and downregulated (green) genes in *svp* RNAi females compared to *Luc* control. (B) Some of the genes significantly enriched based on GO analysis that are up- or downregulated in the absence of *svp*. (C) Quantification of normalized transcript expression (FPKM) for significantly enriched genes in control and *svp* RNAi fat bodies. Data shown as mean±SEM. *P <0.05; **P <0.01; ***P <0.001, two-tailed Student’s *t*-test. (D) Simplified cartoon of detoxification of xenobiotics. Cyp4e3 and Cyp4p2 are predicted to localize to the endoplasmic reticulum (UniProt.org), whereas Cyp12d1-d is predicted to localize to the mitochondrial inner membrane (UniProt.org).

As a second strategy to identify a select group of candidate Svp targets, we also performed GO analysis [clusterProfiler R; (Yu *et al.* 2012)] of transcripts that were significantly altered when *svp* was knocked down in the adult female fat body. In accordance with the analysis described above (of genes with a log2 fold change of at least ±1.5), the GO analysis of our RNA sequencing data did not identify gene enrichment for any specific nutrient or metabolic pathway. Instead, GO analysis showed that a subset of genes involved in the response to xenobiotics (pollutants, insecticides, and drugs) are significantly regulated in response to *svp* knockdown (Figure 4A).

The breakdown of toxic molecules involves an elaborate three-phase system to metabolize xenobiotics into innocuous molecules and facilitate their excretion (Xu *et al.* 2005; Yu 2008). During phase I, xenobiotics are oxidized by cytochrome P450 (Cyp450) monooxygenases to introduce reactive and polar groups to substrates (Yu 2008). These proteins are membrane-bound monooxygenases that are involved in a wide array of physiological processes including detoxification, steroid metabolism, and fatty acid metabolism (Feyereisen 1999; Zangar *et al.* 2004; Xu *et al.* 2005). Xenobiotic metabolites generated during phase I are further converted by phase II enzymes [glutathione-S-transferases (GSTs), UDP-glucuronosyltransferases (UGTs), and carboxylases] through conjugation reactions that add functional side groups (such as hydroxyl, carboxyl, and epoxide) to increase hydrophobicity (Yu 2008). Phase III transporters [ATP-binding cassette (ABC) transporters and Organic anion transport proteins (Oatps)] act in the final phase of detoxification to export converted organic products out of the cell (Groen *et al.* 2017).

Genes involved in each step of the xenobiotic response were altered when *svp* was knocked down in the adult female fat body. Genes encoding members of the Cyp450 family (*Cyp12d1-d*, *Cyp4e3*, and *Cyp4p2*), which initiate phase I of the detoxification process (Xu *et al.* 2005), were significantly downregulated in *svp* knockdown fat bodies (Figure 4B-D). One exception was the gene *phm* (FBgn0004959), encoding a Cyp450 family member involved in ecdysteroid biosynthesis (Chávez *et al.* 2000), which was upregulated in the absence of *svp* (Table S5). Transcription of the phase II enzyme gene, *GstD5* (FBgn0010041), was also significantly decreased in *svp* RNAi fat bodies (Figure 4B,C), suggesting that Svp normally induces expression of these genes. By contrast, genes encoding metallothionein (Mtn) proteins were significantly upregulated in *svp* knockdown fat bodies (Figure 4B-D), suggesting that Svp activity in the adult female fat body represses these genes. Mtn proteins are xenobiotic-induced enzymes involved in heavy metal detoxification and protection against free radicals, and have been implicated in the response to xenobiotic and immune stress (Figure 4D) (Bonneton *et al.* 1996). Additionally, genes encoding detoxification phase III transporters (Oatps) were also upregulated in the fat body in the absence of *svp* (Figure 4B-D; Table S5). Interestingly, *svp* knockdown did not affect the transcript levels of *cap-n-collar* [*cnc* (FBgn0262975); encodes Nrf2 homolog] or *forkhead box, sub-group O* [*foxo* (FBgn0038197)](Table S2), which encode known regulators of detoxifying and antioxidant genes (Ma 2013; Klotz *et al.* 2015). Comparison of known Cnc targets identified by microarray (Misra *et al.* 2011) with those determine by our RNA sequencing analysis identified 22 common transcripts (Table S6), consisting mostly of the detoxifying proteins described above. In addition, comparison of Svp-regulated genes with FOXO targets identified by ChIP-seq (Birnbaum *et al.* 2019) revealed at least 10 common transcriptional targets, including *Cyp4e3* (FBgn0015035) (Table S7). Thus, Svp appears to share some common targets involved in xenobiotic responses with Cnc and Foxo.

It is conceivable that the upregulation of Mtn and Oatp transcripts is a secondary consequence of the downregulation of *Cyp4e3* when *svp* is knocked down in the adult female fat body. It was previously shown that loss of *Cyp4e3* in Malpighian tubules increases the levels of hydrogen peroxide and induces JAK/STAT and NF-κB-mediated stress responses (Terhzaz *et al.* 2015). Mtn transcription is induced in mammalian hepatic cell lines in response to hydrogen peroxide (Dalton *et al.* 1994). Mtns are able to scavenge a variety of reactive oxygen species (ROS), including hydrogen peroxide (Inoue *et al.* 2009), suggesting that Mtns protect against oxidative stress in addition to regulating metal abundance. Therefore, we speculate that *svp* knockdown in the fat body may cause the accumulation of toxic molecules (as a result of *Cyp4e3* downregulation), leading to increased expression of Mtns and Oatps to eliminate these compounds from the cell.

Exposure to xenobiotics has long been known to result in perturbations of the immune system (Luster *et al.* 1989). In addition to their role in metal metabolism, Mtns have also been implicated in immune functions. Specifically, transcription of mammalian hepatocyte MT-1 is activated by STAT binding in response to lipopolysaccharide exposure (Arizono *et al.* 1995; Coyle *et al.* 2002), suggesting that under increased immune challenge Mtns possibly act as antioxidants in response to inflammation (Arizono *et al.* 1995). It is unclear whether *Drosophila* Mtn proteins have roles in regulating the immune response or whether they are induced downstream of the JAK/STAT pathway [potentially also downstream of reduction of *Cyp4e3* (Terhzaz *et al.* 2015)]. In addition, it is unclear whether knockdown of *svp* in adult female fat bodies activates the JAK/STAT pathway. Nevertheless, it would be interesting to investigate whether the increased transcription of *Mtn* and *Oatp* genes observed when *svp* is knocked down might reflect the regulatory cross-talk between Mtns and immune response pathways. Collectively, our RNA sequencing results suggest that loss of *svp* and the reduced expression Cyp450 xenobiotic response genes in the adult female fat body could potentially result in accumulation of toxic compounds, causing activation of secondary downstream stress mechanisms.

**Figure 5:**
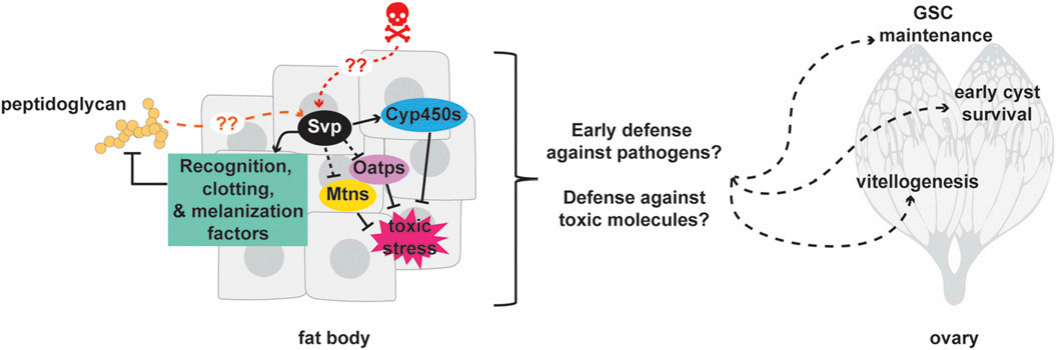
Model for proposed role of Svp in the adult female fat body to regulate oogenesis. Our previous work showed that Svp is required in adult female adipocytes to control GSC number and early germline cyst survival and in oenocytes for vitellogenic follicle survival. This study identifies potential downstream targets for Svp and suggests the model that Svp activity in the fat body might promote oogenesis through a mechanism involving an enhancement of the initial defense against bacteria (to prevent full immune activation) and of Cyp450 detoxification mechanisms (to inhibit secondary stress response pathways).

Oogenesis is an energy-intensive process that is tightly regulated to ensure reproductive success (Schwenke *et al.* 2016; Laws and Drummond-Barbosa 2017). Mounting a full immune response and chemically inactivating harmful toxins from the body also require a significant amount of energy (Buttgereit *et al.* 2000). Our RNA sequencing analysis of potential Svp targets in the adult female fat body suggests that Svp normally functions to: 1) enhance the initial organismal defense against microorganisms and thus prevent a full immune response (*e.g.*, involving the IMD pathway); and 2) optimize the levels of Cyp450s for detoxification of xenobiotics to prevent activation of stress response pathways. Through these actions, Svp might help allocate resources to promote distinct aspects of oogenesis (Figure 5). This model will be tested in future studies, and many additional questions remain regarding this potential model and beyond. For example, it is unknown whether, upon *svp* knockdown, reduced expression of bacterial recognition proteins (and thereby hampered initial bacterial defense) might lead to the activation of the humoral/IMD immune system at a later time point in the fat body. It is also possible that reduced levels of detoxification enzymes in the absence of *svp* might result in the accumulation of xenobiotics that activate an immune response, reminiscent of mammalian innate T-cells that recognize and process allergens and other environmental chemicals prior to their presentation to lymphocytes (Minnicozzi *et al.* 2011; Germolec *et al.* 2017; Maeda *et al.* 2019). It is also not known at this point whether these changes in immune function and xenobiotic response in the absence of *svp* occur in adipocytes, oenocytes, or both cell types, or what their functional consequence is to the steps of oogenesis affected by adipocyte/oenocyte Svp activity (Weaver and Drummond-Barbosa 2019). Finally, it remains to be tested whether the differentially expressed genes identified in our dataset contribute to the regulation of the processes of oogenesis that are affected by *svp* knockdown in adipocytes and oenocytes (Weaver and Drummond-Barbosa 2019). Deciphering the complex mechanisms of Svp activity in the adult female fat body and how they regulate distinct aspects of oogenesis will be a fertile area for future research.
